# Increased Denitrification Rates Associated with Shifts in Prokaryotic Community Composition Caused by Varying Hydrologic Connectivity

**DOI:** 10.3389/fmicb.2017.02304

**Published:** 2017-11-22

**Authors:** Abigail Tomasek, Christopher Staley, Ping Wang, Thomas Kaiser, Nicole Lurndahl, Jessica L. Kozarek, Miki Hondzo, Michael J. Sadowsky

**Affiliations:** ^1^St. Anthony Falls Laboratory, University of Minnesota, Minneapolis, MN, United States; ^2^Department of Civil, Environmental, and Geo-Engineering, University of Minnesota, Minneapolis, MN, United States; ^3^BioTechnology Institute, University of Minnesota, St. Paul, MN, United States; ^4^Water Resources Science, University of Minnesota, St. Paul, MN, United States; ^5^Department of Soil, Water, and Climate, University of Minnesota, St. Paul, MN, United States

**Keywords:** sequencing, bacterial community structure, denitrification, hydrology, qPCR, soil

## Abstract

While modern developments in agriculture have allowed for increases in crop yields and rapid human population growth, they have also drastically altered biogeochemical cycles, including the biotransformation of nitrogen. Denitrification is a critical process performed by bacteria and fungi that removes nitrate in surface waters, thereby serving as a potential natural remediation strategy. We previously reported that constant inundation resulted in a coupling of denitrification gene abundances with denitrification rates in sediments, but these relationships were not maintained in periodically-inundated or non-inundated environments. In this study, we utilized Illumina next-generation sequencing to further evaluate how the microbial community responds to these hydrologic regimes and how this community is related to denitrification rates at three sites along a creek in an agricultural watershed over 2 years. The hydrologic connectivity of the sampling location had a significantly greater influence on the denitrification rate (*P* = 0.010), denitrification gene abundances (*P* < 0.001), and the prokaryotic community (*P* < 0.001), than did other spatiotemporal factors (e.g., creek sample site or sample month) within the same year. However, annual variability among denitrification rates was also observed (*P* < 0.001). Furthermore, the denitrification rate was significantly positively correlated with water nitrate concentration (Spearman's ρ = 0.56, *P* < 0.0001), denitrification gene abundances (ρ = 0.23–0.47, *P* ≤ 0.006), and the abundances of members of the families *Burkholderiaceae, Anaerolinaceae, Microbacteriaceae, Acidimicrobineae incertae sedis, Cytophagaceae*, and *Hyphomicrobiaceae* (ρ = 0.17–0.25, *P* ≤ 0.041). Prokaryotic community composition accounted for the least amount of variation in denitrification rates (22%), while the collective influence of spatiotemporal factors and gene abundances accounted for 37%, with 40% of the variation related to interactions among all parameters. Results of this study suggest that the hydrologic connectivity at each location had a greater effect on the prokaryotic community than did spatiotemporal differences, where inundation is associated with shifts favoring increased denitrification potential. We further establish that while complex interactions among the prokaryotic community influence denitrification, the link between hydrologic connectivity, microbial community composition, and genetic potential for biogeochemical cycling is a promising avenue to explore hydrologic remediation strategies such as periodic flooding.

## Introduction

The expansion of modern agricultural practices and the use of synthetic nitrogen (N) fertilizers have resulted in several environmental and ecological consequences. Approximately, 45% of total fixed nitrogen [as ammonia (NH_3_)] produced annually originates from chemical fertilizers (Canfield et al., 2010), and 50–70% of the fixed nitrogen applied to soils is lost to the atmosphere or through soil leaching (Masclaux-Daubresse et al., 2010). Fixed nitrogen in soils is converted to nitrate (${\text{NO}}_{3^{-}}$) by nitrification, and in tile-drained systems, such as those in the Midwestern United States, the leached ${\text{NO}}_{3^{-}}$ is transported directly to water bodies via tile or field drainage. Nitrate loading leads to eutrophication, decreased dissolved oxygen levels, and negative ecological and health effects (Rabalais et al., 2007; Powlson et al., 2008). Specifically, nitrate export in the Mississippi River contributes to the annual formation of the hypoxic dead zone in the Gulf of Mexico (Rabalais et al., 2007). Anthropogenic alteration of the nitrogen cycle also leads to increased emissions of the greenhouse gas nitrous oxide (N_2_O) through incomplete denitrification (Davidson, 2009; Venterea et al., 2012). While only a small fraction (3–5%) of N applied in fertilizers is lost as N_2_O (Crutzen et al., 2016), this still accounts for 50–60% of global N_2_O emissions (USEPA, 2010). Nitrous oxide gas has a considerably greater global warming potential than other greenhouse gases (Forster et al., 2007).

Nitrate is removed from ecosystems through assimilation into biomass by plants, algae, and microbes or through anaerobic oxidation of ammonia (anammox) processes and microbiologically-driven denitrification (Shapleigh, 2006; Kuenen, 2008). Anammox involves the anaerobic oxidation of ammonium (${\text{NH}}_{4^{+}}$) to nitrogen gas (N_2_), via nitrite (${\text{NO}}_{2^{-}}$) or nitrate (${\text{NO}}_{3^{-}}$), and is carried out by a diverse group of bacteria within the phylum *Planctomycetes* (Kuenen, 2008; Humbert et al., 2010). Denitrification is the step-wise reduction of nitrate to ${\text{NO}}_{2^{-}}$, nitric oxide (·NO), N_2_O, and finally to N_2_. Denitrification is performed by a broad range of prokaryotic species (Shapleigh, 2006) and fungi, and while it is primarily an anaerobic process, it has also been observed in microaerophilic and aerobic environments (Zumft, 1997).

The extent of hydrologic connectivity has been shown to have important ecological impacts related to the transfer of organisms as well as biogeochemical cycling (Pringle, 2003). For example, increased biogeochemical cycling has been demonstrated at the interface between terrestrial and aquatic ecosystems (riparian zones; McClain et al., 2003; Hefting et al., 2006; Wang et al., 2012; Zhu et al., 2013). This results in the formation of “hot spots” of nutrient conversion, including increased rates of denitrification (McClain et al., 2003; Hefting et al., 2006; Wang et al., 2012; Zhu et al., 2013; Tomasek et al., 2017). Organic carbon (Perryman et al., 2011), oxygen concentration (Inwood et al., 2007), as well as soil water content (Pinay et al., 2007), nitrate concentration (Inwood et al., 2007), and water velocity (Arnon et al., 2007; O'Connor and Hondzo, 2008) have been shown to affect denitrification rates among soils. Furthermore, floodplain location and hydrologic connectivity to surface waters significantly impacts denitrification rates (Roley et al., 2012a,b; Mahl et al., 2015; Tomasek et al., 2017).

Previous studies have measured the relationship between physiochemical parameters, denitrification rates, and bacterial community structure (Cao et al., 2008; Harvey et al., 2013; Tatariw et al., 2013; Shrewsbury et al., 2016). However, these studies usually targeted only a few genes in the denitrification pathway. These largely included the gene encoding nitrite reductase (*nirS* or *nirK*), which is specific to denitrifiers (Zumft, 1997), or *nosZ*, encoding nitrous oxide reductase that is important for N_2_O production and shows broad distribution among prokaryotes (Philippot et al., 2011; Domeignoz-Horta et al., 2016). A recent study modeling the effects of physicochemical parameters on N_2_O production found that the addition of nitrification gene abundances, rather than solely soil properties, improved predictive accuracy (Breuillin-Sessoms et al., 2017). Inconsistent trends have been observed relating abundances of denitrification genes with actual process rates (Song et al., 2010; Guentzel et al., 2014; Tomasek et al., 2017), and a broader meta-analysis revealed only a weak correlation between gene abundances and process rates when both were measured (Rocca et al., 2015). These results suggest that the ability to significantly associate gene abundances with process rates may depend upon the specific environment sampled.

Advances in next-generation sequencing technology have allowed for more thorough characterization of prokaryotic communities in the environment (Staley and Sadowsky, 2016). However, due to greater diversity (species richness) in soil and sediment communities, as well as microscale variation in community composition, leveraging a whole community profile to assess potentially functionally relevant shifts remains challenging (Robertson et al., 1997; Blackwood et al., 2006; Schmidt and Waldron, 2015). Thus, in order to determine how community-level variation is related to process rates and functional gene abundances, the three components must be measured simultaneously. We previously reported a relationship between gene abundances and denitrification rates at samples collected from in-channel locations of an agricultural watershed, containing Seven Mile creek, located in the Minnesota River Basin (Tomasek et al., 2017). In contrast, we found limited to no coupling between process rates and gene abundances at intermittently-inundated or never-inundated hydrologic regimes.

In the current study, we expand upon our previous studies relating denitrification rates, physicochemical parameters, and denitrification gene abundances by incorporating prokaryotic community compositions, determined using Illumina next-generation sequencing of the V4 hypervariable region of the 16S rRNA gene. Samples were collected from three sites in the agriculturally-dominated Seven Mile Creek (SMC) watershed over 2 years. The hydrologic connectivity of sampling locations varied over the course of the study (ranging from constantly inundated to never inundated) due to large differences in precipitation as well as sample location. We hypothesized that the hydrologic connectivity of a sampling location would have a greater influence on denitrification rates, gene abundances, and bacterial communities than variation in sampling date and site location in SMC. Furthermore, we suspected that community composition would be associated with denitrification rates due to the relatively broad distribution of denitrification genes. Results of this study reveal how varying hydrologic connectivity affects denitrification rates and further provide novel information regarding the interaction and influence of the prokaryotic community at large on denitrification rates resulting from these hydrologic conditions.

## Materials and methods

### Sample collection

Soil samples were collected from three sites in the Seven Mile Creek (SMC) watershed, located in the Minnesota River basin in southern Minnesota (Tomasek et al., 2017). Sites SMC1 (44.2925 N, 94.0759 W) and SMC2 (44.3117 N, 94.0614 W) are located in an agricultural ditch and are surrounded by predominantly agricultural land use, and SMC3 (44.2633 N, 94.0320 W) is located in a nearby county park (Figure S1). At SMC1 and SMC2, three positions with differing hydrologic connectivity were sampled along a transect that included the channel, which was constantly inundated, the floodzone, which was periodically inundated, and the nonfloodzone which was in the riparian region, but never inundated. Samples were only collected from the channel position at SMC3.

Samples were collected on June 12, August 20, and October 20 in 2014 and on May 12, June 15, July 27, August 18, and November 9 in 2015. Triplicate samples were collected for DNA extraction, within 5 cm of each other, using a 5 ml syringe with the top removed. Samples were immediately stored on dry ice, and held at −80°C prior to extraction. Triplicate samples were collected using a 35 ml syringe for measurement of soil nitrate, bulk density, soil organic matter, and moisture content. For measurement of denitrification rates, a 60 ml syringe was used to collect soil to a depth of 5 cm (Inwood et al., 2007). Soil cores were transferred to plastic bags, transported on ice, stored at 4°C, and denitrification rates were determined within 2 days of collection (Findlay et al., 2011). Water width and depth were also measured, as was water velocity using a SonTek Flowtracker (Xylem, Inc., Rye Brook, NY, USA). Triplicate, 1 L, water samples were also collected to determine nitrate concentrations.

### Determination of physicochemical parameters

Environmental parameters, including nitrate concentration, were measured as described previously (Tomasek et al., 2017). Water samples were filtered through pre-combusted 0.7 μm GF/F filters (Whatman, Marlborough, MA, USA). Nitrate concentrations were determined using the cadmium reduction method on a Lachat QC800 Autoanalyzer (Hach Company, Loveland, CO, USA). Shear velocities were calculated from the time-averaged velocity at varying water depths (Biron et al., 2004). Bulk density and moisture content were determined by drying soil cores for 24 h at 110°C and normalizing the difference between dry and wet weight by soil volume. Soil organic matter was determined using the loss on ignition method (LOI), where dried soil was passed through a 2 mm sieve and approximately 5 g was burned at 550°C for 4 h (Heiri et al., 2001). Soil nitrate was measured through water extractions, where the nitrate concentration of the extracted water was measured as described above and the concentration was normalized by the dry soil weight of the sample.

Denitrification rates were determined using a modified acetylene block method (Groffman et al., 2006; Loken et al., 2016; Tomasek et al., 2017). To determine unamended denitrification potential (DN_U_), site-specific water samples were treated with 10 mg L^−1^ chloramphenicol. N_2_O concentrations were analyzed using a 5890 series II gas chromatograph (Hewlett-Packard Enterprise, Palo Alto, CA, USA) equipped with an electron capture detector and headspace autosampler (Hewlett-Packard, 7694) as described previously (Loken et al., 2016). Accumulation of N_2_O over the incubation period was corrected using the Bunsen solubility coefficient (Tiedje, 1982). Denitrification rates were calculated as a function of bulk density and reported as an areal rate (Tomasek et al., 2017). Amended denitrification rates were determined as described previously (Tomasek et al., 2017) following amendment with nitrate (100 mg N L^−1^ as potassium nitrate), carbon (40 mg C L^−1^ as glucose), and phosphate (13.84 mg P L^−1^ as potassium dihydrogen phosphate).

### Denitrification gene abundances

DNA was extracted from triplicate 500 mg soil samples using the DNeasy PowerSoil Kit (QIAGEN, Hilden, Germany) according to the manufacturer's instructions, and DNA was used as template for quantitative PCR (qPCR) assays to determine gene abundances, as described previously (Tomasek et al., 2017). Denitrification gene abundances were determined for *narG* (encoding nitrate reductase, primers narG-1960m2fE/narG2050m2R; Kandeler et al., 2006), *nirK* (encoding nitrite reductase, primers nirK876F/1040R; Petersen et al., 2012), *nirS* (encoding nitrite reductase, primers m-cd3AF/m-R3cd; Petersen et al., 2012), c*norB* (encoding nitric oxide reductase, primers cnorB-BF/cnorB-BR; Dandie et al., 2007), *nosZ1* (encoding nitrous oxide reductase, primers nosZ_F/nosZ_1622R; Rösch et al., 2002), and *nosZ3* (encoding nitrous oxide reductase, primers nosZ2F/nosZ2R; Petersen et al., 2012). Laboratory triplicates were run for all field triplicate samples. Standard curves were generated using gBlocks® Gene Fragments (Integrated DNA Technologies, Inc., Coralville, IA, USA; Svec et al., 2015; Tomasek et al., 2017), except for *nirS*, for which a plasmid standard was used. Tests for inhibition were not performed, and efficiencies for all targets ranged from 80 to 110 with *r*^2^ > 0.99. Negative, no-template controls were included with each qPCR run. Gene abundances were normalized per g dry soil for analysis.

### Illumina sequencing and bioinformatics

The V4 hypervariable region of 16S rRNA was amplified from triplicate DNA extracts per sample using the 515F/806R primer set (Caporaso et al., 2012) and sequenced using the dual index method by the University of Minnesota Genomics Center (UMGC, Minneapolis, MN, USA) (Gohl et al., 2016). Samples from 2014 were paired-end sequenced on the Illumina HiSeq2500 (Illumina, Inc., San Diego, CA, USA) at a read length of 150 nucleotides (nt) and samples from 2015 were paired-end sequenced on the Illumina MiSeq at a read length of 300 nt. The use of different sequencing platforms has been previously shown not to bias biological conclusions drawn (Caporaso et al., 2012). Raw data are deposited in the Sequence Read Archive of the National Center for Biotechnology Information under BioProject accession number SRP113317.

Sequence data were processed and analyzed using mothur version 1.35.1 (Schloss et al., 2009), as described previously for V6 and V5V6 hypervariable regions (Staley et al., 2015b). Samples were trimmed to 150 nt and paired-end joined using fastq-join software with an average join length of approximately 10 nt (Aronesty, 2013), trimmed for quality based on quality score (>35 over a 50 nt window), ambiguous bases (0), homopolymer length (≤8), and primer mismatches (≤2). High quality sequences were aligned against the SILVA database version 123 (Pruesse et al., 2007), subjected to a 2% pre-cluster (Huse et al., 2010), and UCHIME was used to remove chimeric sequences (Edgar et al., 2011). Following quality trimming, the average sequence length was approximately 174 nt. For statistical comparisons, all samples were rarefied by random subsampling to 38,000 sequence reads per sample to reduce statistical bias due to varying numbers of sequences reads (Gihring et al., 2012). Operational taxonomic units (OTUs) were assigned at 97% similarity using complete-linkage clustering and taxonomic classifications were assigned against the Ribosomal Database Project, version 14 (Cole et al., 2009). Different databases were used for alignment and OTU classification due to considerations described previously (Schloss, 2009).

### Statistical analyses

ANOVA analyses with Tukey's *post-hoc* test, Spearman rank correlations, and canonical correspondence analysis were performed using XLSTAT version 2015.6 (Addinsoft, Belmont, MA, USA). Shannon indices, beta diversity calculations, and ordination plots were calculated using mothur. Beta diversity analysis and ordination were performed using Bray-Curtis dissimilarity matrices (Bray and Curtis, 1957). Differences in community composition were evaluated by analysis of molecular variance (ANOSIM; Clarke, 1993) and sample clustering was evaluated by analysis of molecular variance (AMOVA; Excoffier et al., 1992). Ordination was performed by principal coordinate analysis (PCoA; Anderson and Willis, 2003) and Spearman correlations of family abundances associated with ordination were calculated using the corr.axes command in mothur. Variance partitioning was performed by partial redundancy analysis using the vegan package in R, as described previously (Borcard et al., 1992; R Core Team, 2012; Oksanen et al., 2015). All statistics were calculated at α = 0.05 with Bonferroni correction for multiple comparisons.

## Results

### Denitrification rates and physicochemical parameters

Among samples collected at channel locations from all sites (those constantly inundated), large differences were observed among hydrologic parameters (shear velocity, discharge, and depth), both unamended denitrification potential (DN_U_) as well as denitrification potential following amendment with nutrients (DN_A_), and nitrate (${\text{NO}}_{3^{-}}$) concentrations over the study period (Table 1). Hydrologic parameters were greater in 2015 (0.024 ± 0.021 m s^−1^, 0.45 ± 0.24 m, and 0.89 ± 1.1 m^3^ s^−1^, respective to shear velocity, depth, and discharge (Figure S2) than those observed in 2014 (0.009 ± 0.009 m s^−1^, 0.34 ± 0.23 m, 0.28 ± 0.37 m^3^ s^−1^) at all sites. Similarly DN_U_ was greater in 2015 (16.48 ± 20.47 mg N m^−2^ h^−1^ vs. 5.55 ± 9.39 N m^−2^ h^−1^ in 2014) than in 2014, while DN_A_ was significantly greater at the SMC2 site during both years of sampling (Table 1). Furthermore, water discharge was significantly and positively correlated with ${\text{NO}}_{3^{-}}$ concentration (Spearman's ρ = 0.690, *P* < 0.0001) and DN_u_ (ρ = 0.389, *P* = 0.001), and DN_u_ was also positively correlated with ${\text{NO}}_{3^{-}}$ (ρ = 0.561, *P* < 0.0001) and DN_A_ (ρ = 0.636, *P* < 0.0001).

**Table 1:** Hydrologic parameters, nitrate concentration, and denitrification potential among channel sites.

**Year**	**Site**	***n***	**Shear velocity (m s^−1^)**	**Discharge (m^3^ s^−1^)**	**Depth (m)**	**NO_3_ as N (mg L^−1^)**	**DN_U_ (mg N m^−2^ h^−1^)**	**DN_A_ (mg N m^−2^ h^−1^)**
2014	SMC1	9	0.004 ± 0.000	0.28 ± 0.4	0.57 ± 0.05	13.51 ± 20.24	7.70 ± 14.64	15.24 ± 13.03
	SMC2	9	0.003 ± 0.004B		0.31 ± 0.24B	11.43 ± 16.88	4.29 ± 6.14B	28.69 ± 9.77AB
	SMC3	9	0.018 ± 0.010B		0.13 ± 0.02B	13.16 ± 10.69	4.66 ± 5.20B	5.49 ± 6.37B
2015	SMC1	15	0.012 ± 0.009B	0.89 ± 1.1	0.64 ± 0.08A	17.18 ± 10.81	14.23 ± 11.79B	24.48 ± 15.83B
	SMC2	15	0.016 ± 0.009B		0.52 ± 0.23A	17.79 ± 11.29	30.16 ± 27.77A	47.79 ± 40.22A
	SMC3	15	0.043 ± 0.025A		0.18 ± 0.07B	14.00 ± 5.43	4.24 ± 4.76B	5.30 ± 5.45B
Fisher's F^*^			<0.0001	0.196	<0.001	0.796	<0.001	<0.001

*Values as mean ± standard deviation are shown, and n represents the number of samples for each site. Data from site SMC1 in 2014 were excluded from analyses due to multicolinearity*.

* *Fisher's F statistics for the ANOVA model. Separate models were calculated for each variable*.

*^A, B^Values sharing the same letter did not vary significantly by Tukey's post-hoc test (P > 0.05)*.

**Figure 1:**
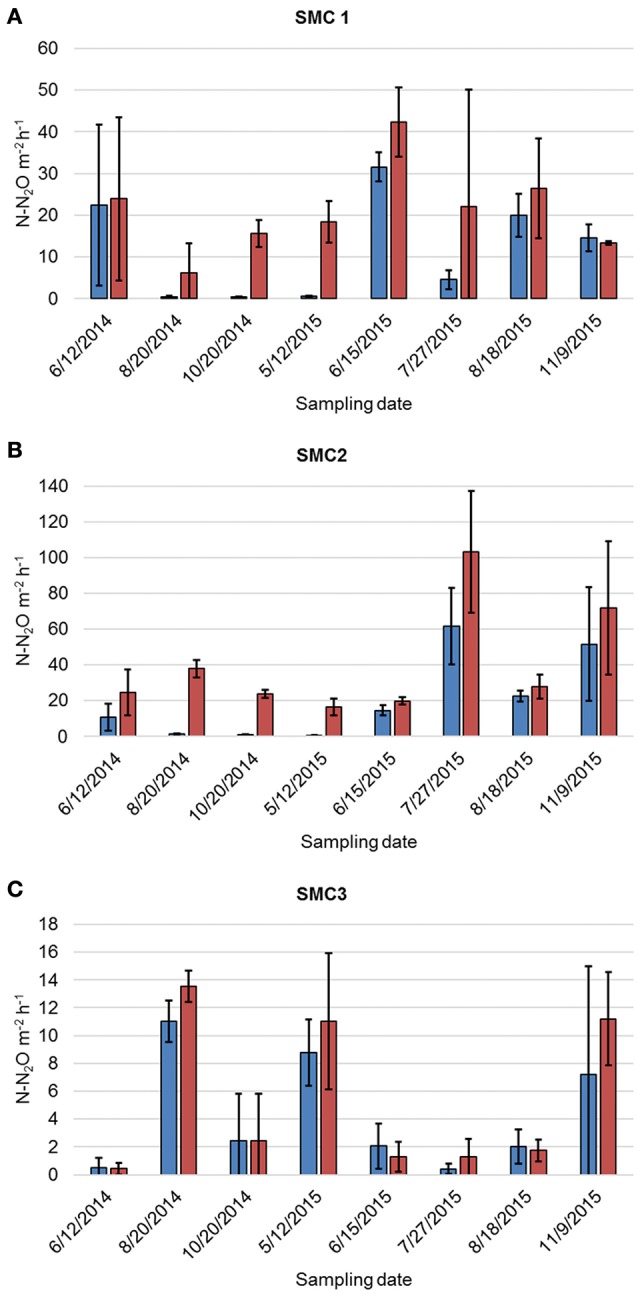
Mean denitrification rates of triplicate samples collected from channel positions at **(A)** SMC1, **(B)** SMC2, and **(C)** SMC3. Error bars reflect standard deviation. Blue bars represent DN_U_ and red bars represent DN_A_.

Denitrification rates varied between sampling years, as well as among sampling months within the same year, spatial position (i.e., channel, floodzone, or nonfloodzone), and sampling site (ANOVA *P* < 0.0001, Figure 1, Tables S1, S2). The unamended denitrification rate (DN_U_) was significantly greater in 2015 than in 2014 (Tukey's *post-hoc P* < 0.0001), greater among channel samples than samples collected in the nonfloodzone (*P* = 0.010), and greater at SMC2 than the other sites (*P* < 0.001). Similarly, DN_A_ was greater in 2015 (*P* = 0.005), greater among channel samples than those from the nonfloodzone (*P* = 0.028), and significantly different among all sites in the order of: SMC2 > SMC1 > SMC3 (*P* < 0.001). In addition, physicochemical measurements, including the dry weight:wet weight ratio, volumetric water content, bulk density, organic matter, and soil nitrate (Table S3), also showed significant variation among sampling sites and positions, but showed less temporal variability.

### Denitrification gene abundances

**Figure 2:**
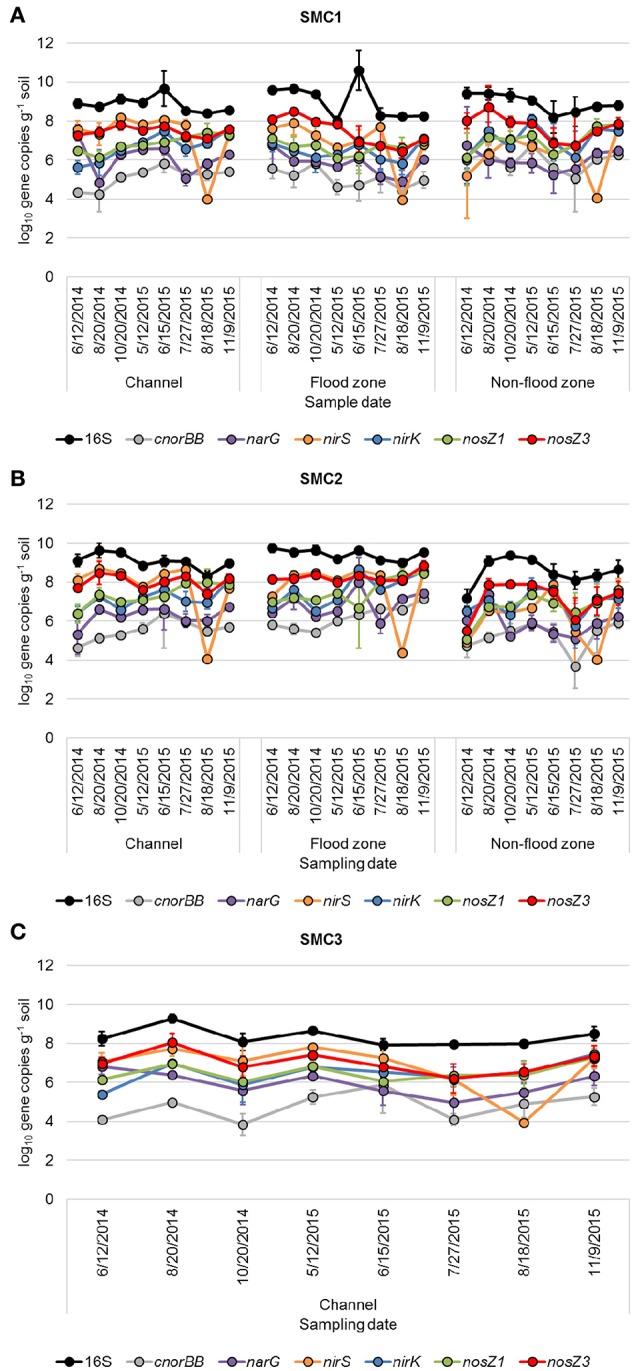
16S rRNA and denitrification gene abundances from samples collected at **(A)** SMC1, **(B)** SMC2, and **(C)** SMC3. Error bars reflect standard deviation of triplicate samples.

Gene abundances quantified by qPCR (Figure 2) were significantly different due to variability in time, hydrologic connectivity, and site location along SMC (ANOVA Fisher's *F* < 0.0001 for all genes). The 16S rRNA gene abundances were significantly greater in 2014 compared to 2015 (Tukey's *P* = 0.008), at the floodzone relative to the nonfloodzone (*P* < 0.001), and at SMC1 and SMC2 relative to SMC3 (*P* < 0.001). Among denitrification genes, significantly lower relative abundances of genes were also typically observed in nonfloodzone samples (Table 2).

**Table 2:** Differences in denitrification gene abundances due to temporal, spatial, and geographic features.

**Gene**	**Year**	**Location**	**Site**
*cnorBB*	2015 > 2014 (0.020)	NS^*^	SMC2 > SMC3 (0.005)
*narG*	2015 > 2014 (0.010)	floodzone > nonfloodzone (0.037)	SMC2 > SMC3 (0.012)
*nirS*	NS	nonfloodzone < others (< 0.001)	SMC2 > SMC1 > SMC3 (≤ 0.035)
*nirK*	2015 > 2014 (< 0.0001)	NS	SMC2 > others (< 0.001)
*nosZ1*	2015 > 2014 (< 0.0001)	NS	SMC2 > SMC1 > SMC3 (≤ 0.039)
*nosZ3*	NS	Nonfloodzone < others (≤ 0.047)	SMC2 > SMC1 > SMC3 (≤ 0.032)

*Tukey's post-hoc P values are shown in parentheses, where significant (P < 0.05). Only the abundances of cnorBB did not show some significant variation among months of sampling (P ≥ 0.105)*.

* *NS: no significant differences were observed in relation to the variable*.

Abundances of all genes except *nirS* were significantly and positively correlated with DN_U_ (ρ = 0.231–0.469, *P* ≤ 0.006), and the abundances of all genes were significantly correlated with DN_A_ (ρ = 0.325–0.516, *P* < 0.0001), among all samples. When samples were analyzed by sampling position, among both years and all samples sites (Table S4), significant positive correlations among denitrification rates and gene abundances were only found among samples collected from the channel and floodzone positions, with one exception. Furthermore, these correlations were generally stronger (ρ ≥ 0.490) than those found when samples from all positions were combined.

### Alpha diversity and community composition

The number of OTUs varied from 385 to 8,160 OTUs per sample, with a mean Good's coverage of 96.3 ± 0.3%, among all samples. Samples collected in 2014 had significantly lower alpha diversity, measured by the Shannon index, compared to those collected in 2015 (mean 4.07 ± 0.23 in 2014 and 7.26 ± 0.40, *P* < 0.0001). Among samples collected in 2014 (Figure S3A), Shannon indices among samples collected at SMC2 were significantly greater than those at SMC3 (Tukey's *P* = 0.010), and diversity decreased by position as: channel > floodzone > nonfloodzone (*P* ≤ 0.002). Similar patterns were observed in 2015 (Figure S3B), except there was no significant difference between samples collected in the channel and floodzone (*P* = 0.546) and samples collected in July and August had significantly greater diversity than those collected in May (*P* = 0.013 and 0.018, respectively).

**Figure 3:**
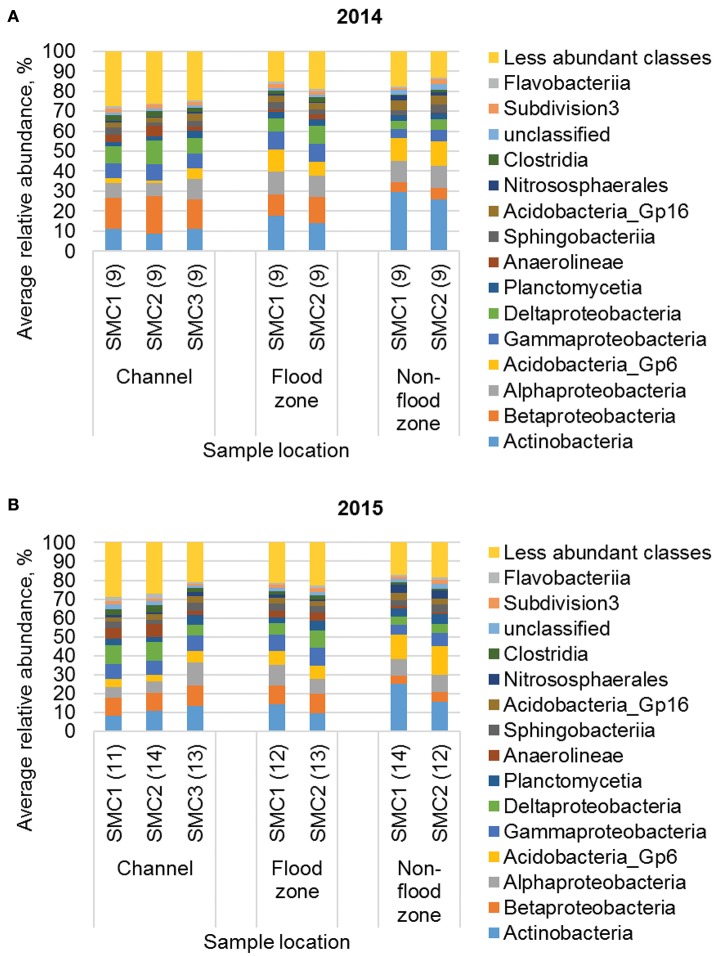
Distribution of the 15 most abundant classes from samples collected in **(A)** 2014 and **(B)** 2015. Numbers in parentheses reflect sample size. Samples in 2015 that did not meet the rarefaction depth of 38,000 sequence reads were excluded from the dataset. Less abundant classes were present at a mean ≤1.6% of sequence reads, among all samples.

During both years of sampling, communities from all sampling sites were predominantly comprised of members of the Actinobacteria, α- and β-Proteobacteria, and Acidobacteria classes (Figure 3). Family-level classification (Figure S4) revealed that communities were predominantly comprised of many low-abundance families, and approximately 20–30% of sequence reads could not be assigned at this taxonomic level.

### Beta diversity

Prokaryotic community composition (beta diversity) among samples differed significantly between sampling years (ANOSIM R = 1.000, *P* < 0.001, Figure S5). During both sampling years (taken together), differences in beta diversity varied significantly, more due to hydrologic connectivity associated with sample positions (*R* = 0.271, *post-hoc P* < 0.001) than to sampling sites (*R* = 0.066, *P* ≤ 0.013). These trends were maintained when individual sampling years were evaluated separately. Moreover, prokaryotic communities did not vary significantly by sampling month in either year (*r* = 0.031, *P* = 0.117 and *r* = −0.004, *P* = 0.498, in 2014 and 2015, respectively).

**Figure 4:**
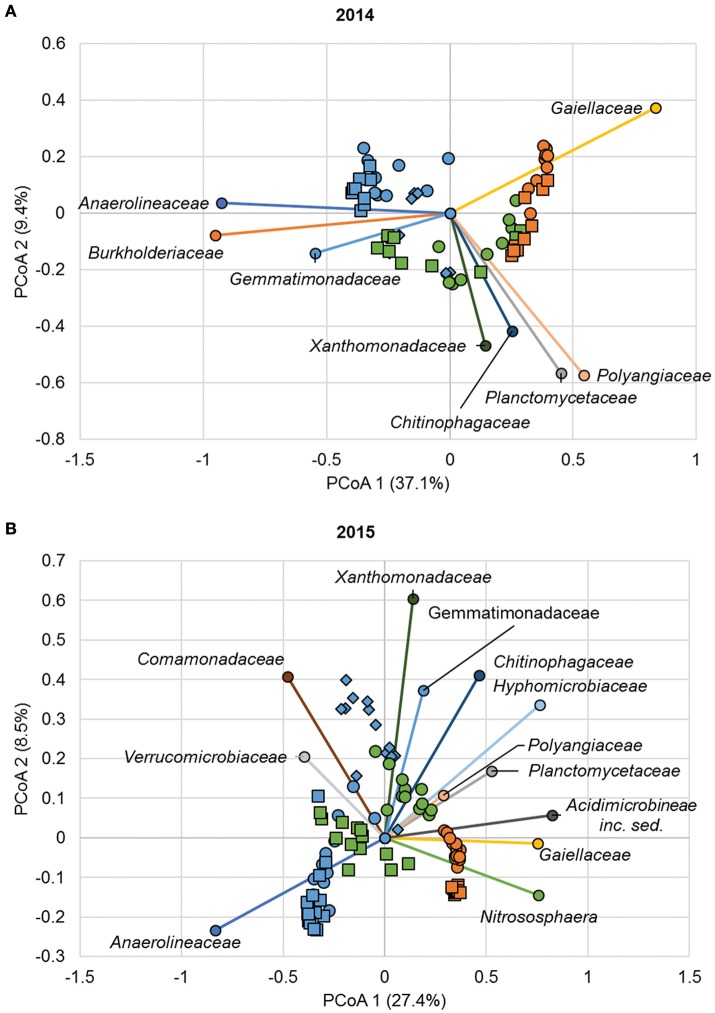
Principal coordinate analysis of Bray-Curtis dissimilarity matrices from samples collected in **(A)** 2014 (*r*^2^ = 0.82) and **(B)** 2015 (*r*^2^ = 0.73). Legend: SMC1 (•), SMC2 (■), SMC3 (♦), channel (blue), floodzone (green), nonfloodzone (orange). Relative abundances of families shown (among the 15 most abundant shown in Figure S4) were significantly correlated with ordination position (*P* < 0.05). Families that were not significantly correlated are not shown.

Similar to ANOSIM results, ordination of Bray-Curtis dissimilarity matrices by PCoA revealed clustering of samples primarily based on sample position (AMOVA *F*_*s*_ = 19.8, *P* < 0.001 and *F*_*s*_ = 16.8, *P* < 0.001 for 2014 and 2015, respectively; Figure 4). Spearman correlation analyses relating family abundances to ordination position revealed similar trends during both years of sampling (*P* ≤ 0.006; Figure 4). For example, abundances of members of the family *Anaerolineaceae* were significantly related to samples collected from the channel, as indicated by similar ordination position. Similarly, members of the families *Cytophagaceae, Gemmatimonadaceae*, and *Xanthomonadaceae* were generally associated with floodzone samples, and the *Gaiellaceae* were found at greater abundances in nonfloodzone samples.

### Associations among prokaryotic communities and denitrification

Variance partitioning was performed using constrained redundancy analyses to determine how the prokaryotic community influenced denitrification rates (DN_U_ and DN_A_) in conjunction with temporal, spatial, physicochemical parameters, and denitrification gene abundances. By using this method, the prokaryotic community composition alone, taken as abundances of predominant families (those present at a mean of at least 1.0% of sequence reads), accounted for 21.8% of variation in denitrification rates (DN_U_ and DN_A_). Non-community factors (including abundances of denitrification genes) accounted for 37.1% of the variation in denitrification rates, and interactions among all parameters accounted for 41.1%.

**Figure 5:**
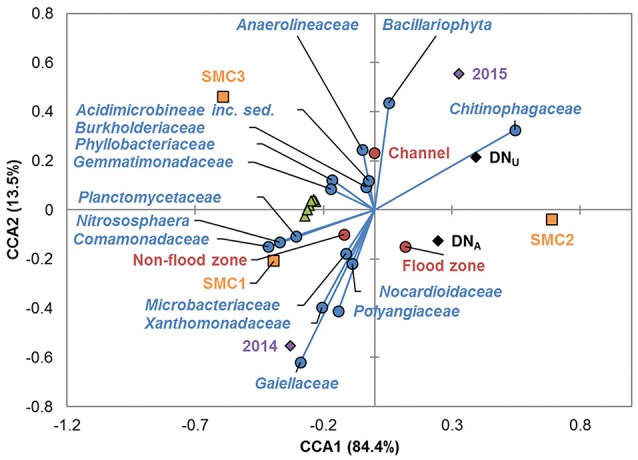
Canonical correspondence analysis of sampling sites, years, and positions; denitrification rates; denitrification gene abundances; and family abundances (among the 10 most abundant). Legend: sampling sites (■), years (♦), positions (•), denitrification rates (■), denitrification genes (▴), and prokaryotic families (•).

The relationships among all parameters modeled were further evaluated by canonical correspondence analysis (CCA; Figure 5). Denitrification gene abundances were found to cluster closely and were similarly all significantly positively inter-correlated by Spearman correlation (ρ = 0.221–0.826, median = 0.452, *P* ≤ 0.004). Similarly, abundances of bacterial families were found to associate with channel and nonfloodzone features, as observed in the PCoA analyses (above). In contrast, families associated with the floodzone by PCoA showed inconsistent relationships by CCA. No consistent trends were observed for correlations among family abundances and denitrification genes (e.g., abundances of the archaeal nitrifier *Nitrososphaera* were not significantly correlated with gene abundances by traditional Spearman correlation). Abundances of members of the families *Burkholderiaceae, Anaerolinaceae, Microbacteriaceae, Acidimicrobineae incertae sedis, Cytophagaceae*, and *Hyphomicrobiaceae* (in order of abundance) were positively correlated with DN_U_ (ρ = 0.169–0.251, *P* ≤ 0.041), while the abundances of members of the families *Gaiellaceae, Comamonadaceae*, and *Sinobacteraceae* were negatively correlated (ρ = −0.291 to −0.168, *P* ≤ 0.042).

## Discussion

In this study, denitrification rates under site conditions (DN_u_) and under non-limiting nutrient concentrations (DN_A_) varied in response to spatiotemporal parameters as well as hydrologic connectivity. Differences between DN_U_ and DN_A_ rates can provide insight into environmental parameters limiting denitrification under field conditions, and allow for comparisons of the potential of sites to denitrify under optimal conditions. Under unfavorable field conditions, for instance low nitrate concentrations, denitrification will be limited even if denitrifying bacteria are present. Therefore DN_A_ rates are valuable when correlating denitrification rates and gene abundances. The measured DN_u_ rates were similar to those found in previous studies in agricultural systems (Roley et al., 2012a; Mahl et al., 2015; Tomasek et al., 2017). In August and October 2014, and May 2015, when nitrate concentrations were lowest, the low DN_u_ and the large differences between DN_u_ and DN_A_ implied that channel locations at SMC1 and SMC2 were likely nitrate-limited on these dates. At the nonfloodzone locations of both SMC1 and SMC2, DN_U_ and DN_A_ remained relatively constant throughout the study period. SMC3 had much lower DN_U_ and DN_A_ compared to SMC1 and SMC2 channel locations, which may be due to site characteristics including sandy sediment and greater shear velocities, leading to a potentially less stable microbial community due to increased bedload transport (Arnon et al., 2007; Tomasek et al., 2017). This supposition is further supported by the significantly lower abundances of the 16S rRNA gene and the next-generation sequencing data, where significantly lower diversity was observed at SMC3 relative to SMC2.

Previous research has shown that reconnecting channels with riparian areas can enhance denitrification (Kaushal et al., 2008; Klocker et al., 2009; Roley et al., 2012a; Mahl et al., 2015). The two agricultural sites in this study, SMC1 and SMC2, had differing ditch geometry. SMC1 had a traditional trapezoidal configuration, whereas SMC2 had an inset depositional floodplain at the floodzone location (Tomasek et al., 2017). Therefore, the floodzone location at SMC2 would have a larger reactive surface area, more sediment-water contact time, a larger hyporheic zone, and would likely favor greater rates of nitrogen cycling (McClain et al., 2003; Hefting et al., 2006; Wang et al., 2012; Woodward et al., 2015). Precipitation during the summer of 2014 occurred largely in one rain event in late June, whereas the rest of the summer was relatively dry. In comparison, precipitation was greater and occurred more frequently throughout the summer in 2015. The increased precipitation in 2015 caused inundation at the floodzone location, particularly at SMC2. This likely caused the differential correlations between denitrification rates, environmental parameters, and gene abundances in 2015 compared to 2014 at floodzone locations. The floodzone location at SMC2 had significantly greater soil moisture content and DN_u_ in 2015 than in 2014. However, there was no significant difference between 2015 and 2014 in soil water content or DN_U_ at the floodzone location of SMC1. Denitrifying gene abundances were also significantly greater at the floodzone location of SMC2 compared to SMC1 in 2015.

Abundances of nearly all denitrification genes investigated were significantly and positively correlated with DN_U_ and DN_A_, suggesting that, in this ecosystem, these abundances are related to active transcription and process rates (Rocca et al., 2015). We previously reported the gene abundances were coupled with denitrification rates at only channel positions among 2014 samples (Tomasek et al., 2017). In the current study, when samples were grouped by hydrologic regime, denitrification rates were significantly correlated with gene abundances for the channel and floodzone positions; however, there was only one significant correlation between *nirS* and DN_A_ among nonfloodzone samples. This may explain why similar denitrification rates were observed across sampling dates at the nonfloodzone locations, where unfavorable environmental conditions limit denitrification rates, even when denitrifying bacteria are present. Our data may suggest that inundation is one method that may induce a denitrification response by providing more favorable environmental conditions (Tomasek et al., 2017). Furthermore, a previous study similarly suggested that flooding induced a physiological response among denitrifiers (Manis et al., 2014).

Legacy effects associated with soil moisture have recently been shown to significantly impact the microbial community composition and N_2_O flux (Banerjee et al., 2016). While early inundation shaped a community favoring *Burkholderiaceae* in 2014, only a short-term increase in denitrification was observed in June, while a more sustained response was observed during 2015, when soil moisture and precipitation were greater throughout the sampling period. Recent work has similarly suggested that hydroecology during Spring is likely to help shape the microbial community (Esposito et al., 2016). However, samples between years were also sequenced on different instruments. While differences in sequencing platform may have had minor effects on the communities characterized, results using different sequencing platforms have previously been shown to be comparable (Caporaso et al., 2012), especially among environmental samples processed using a pipeline similar to that used in this study (Staley et al., 2015a).

Inclusion of next-generation bacterial community characterization further elucidates how microbial community structure changed as a result of hydrologic connectivity, and how this community influenced denitrification rates. Soil microbial communities have previously been shown to vary more as a result of site than specific treatments (Fernandez et al., 2016a,b). Here we also reported differences in community composition between sampling years as well as between sampling sites, but, interestingly, the hydrologic regime more strongly drove differences in community composition than did geographic variation. Similar to a previous report (Argiroff et al., 2017), increased hydrologic connectivity corresponded with an increase in the abundances of Proteobacteria and significantly separated communities characterized by both family-level abundances, as well as functional gene categories. Not surprisingly, more frequent inundation favored more highly anaerobic communities, but genes associated with denitrification were more evenly spread across three soils with varying hydrologic connectivity (Argiroff et al., 2017). We found few correlations between family-level abundances and either denitrification rates or gene abundances, which is not surprising given the functional redundancy resulting from the wide distribution of these genes (Zumft, 1997; Shapleigh, 2006). Furthermore, prokaryotic community composition and its interaction with other spatiotemporal and physicochemical parameters caused a considerable amount of variation in denitrification rates. Several of the families correlated with denitrification rate were significantly associated with inundation, such as *Anaerolinaceae* and *Microbacteriaceae*, suggesting these families potentially play an important role in denitrification.

## Conclusions

Results of this study reveal how varying hydrologic regimes associated with differences in hydrologic connectivity influence both denitrification rates as well as prokaryotic community composition. Frequent inundation increases both denitrification gene abundances and denitrification rates, and indirectly influences the composition of the microbial community. Abundances of the families *Burkholderiaceae, Anaerolinaceae, Microbacteriaceae, Acidimicrobineae incertae sedis, Cytophagaceae*, and *Hyphomicrobiaceae* were significantly correlated with DN_U_ and may be among the most active in denitrification under the varying hydrological conditions tested. Further study is necessary to determine which environmental parameters are most likely to shift microbial communities to stimulate biogeochemical processes including denitrification. However, this study provides novel evidence that inundation drives shifts in the microbial community that increase denitrification rates. Thus, changing patterns of hydrologic connectivity, for example by periodic flooding, may serve as an effective management strategy to remediate nitrate pollution by causing corresponding shifts in the microbial community.

## Author contributions

AT, CS, MH, JK, and MS help conceive and guide the study. AT, CS, PW, TK, and NL helped carry-out experiments. AT, CS, JK, MH and MS helped write the manuscript.

### Conflict of interest statement

The authors declare that the research was conducted in the absence of any commercial or financial relationships that could be construed as a potential conflict of interest. The reviewer PV and handling Editor declared their shared affiliation.
