# Supplementary material for: Increased Denitrification Rates Associated with Shifts in Prokaryotic Community Composition Caused by Varying Hydrologic Connectivity
Source: Front Microbiol. 2017 Nov 22;8:2304. doi: 10.3389/fmicb.2017.02304 (PMC5702768; doi:10.3389/fmicb.2017.02304)
Supplement: Supplementary file 1 [file Presentation1.PDF]

Supplemental Figures and Tables

Increased Denitrification Rates Associated with Shifts in Prokaryotic Community Composition Caused by Varying Hydrologic Connectivity

Abigail Tomasek[1,2,*], Christopher Staley[3,*], Ping Wang[3], Thomas Kaiser[3], Nicole Lurndahl[4], Jessica Kozarek[1], Miki Hondzo[1,2], and Michael J. Sadowsky[3,5,#]

[1]St. Anthony Falls Laboratory, University of Minnesota, Minneapolis, 55455, United States
[2]Department of Civil, Environmental, and Geo-Engineering, University of Minnesota, Minneapolis, 55455, United States
[3]BioTechnology Institute, University of Minnesota, St. Paul, 55108, United States
[4]Water Resources Science, University of Minnesota, St. Paul, 55108, United States
[5]Department of Soil, Water, and Climate, University of Minnesota, St. Paul, 55108, United States

[*]Authors share first authorship

Running title: Denitrification rates and prokaryotic communities

[#]Corresponding Author: Michael J. Sadowsky, BioTechnology Institute, University of Minnesota, 140 Gortner Lab, 1479 Gortner Ave, Saint Paul, MN 55108; Phone: (612)-624-2706, Email: sadowsky@umn.edu

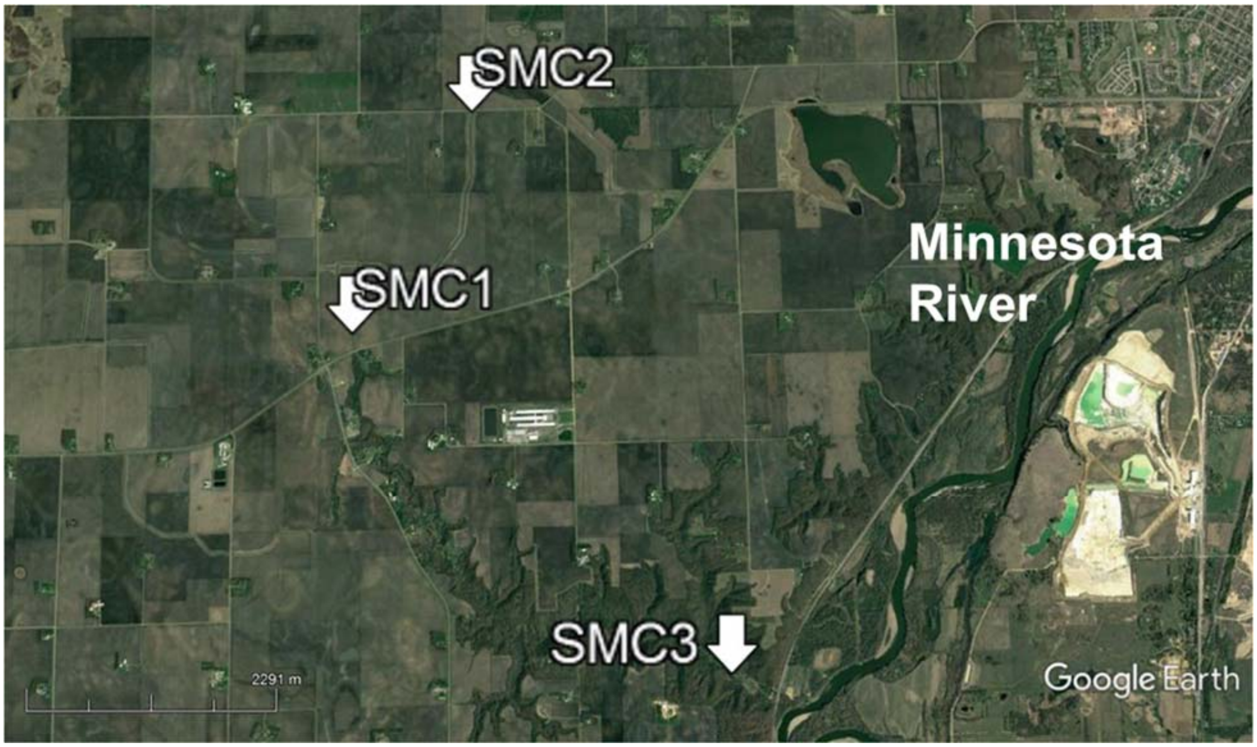

Figure S1. Map of sampling sites. The map was generated using Google Earth.

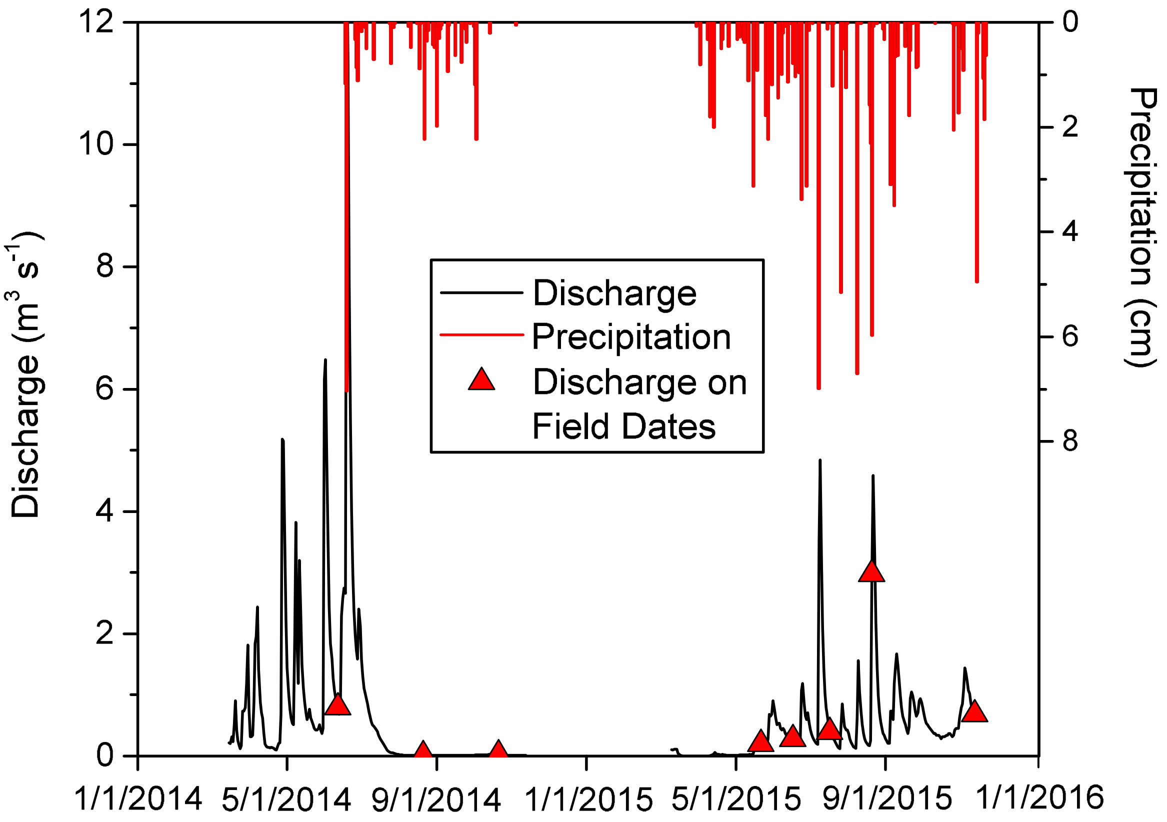

Figure S2. Discharge (black) and precipitation (red) recorded at SMC3 in the Seven Mile Creek Watershed over the duration of the study period. Red triangles represent the discharge on the sampling dates.

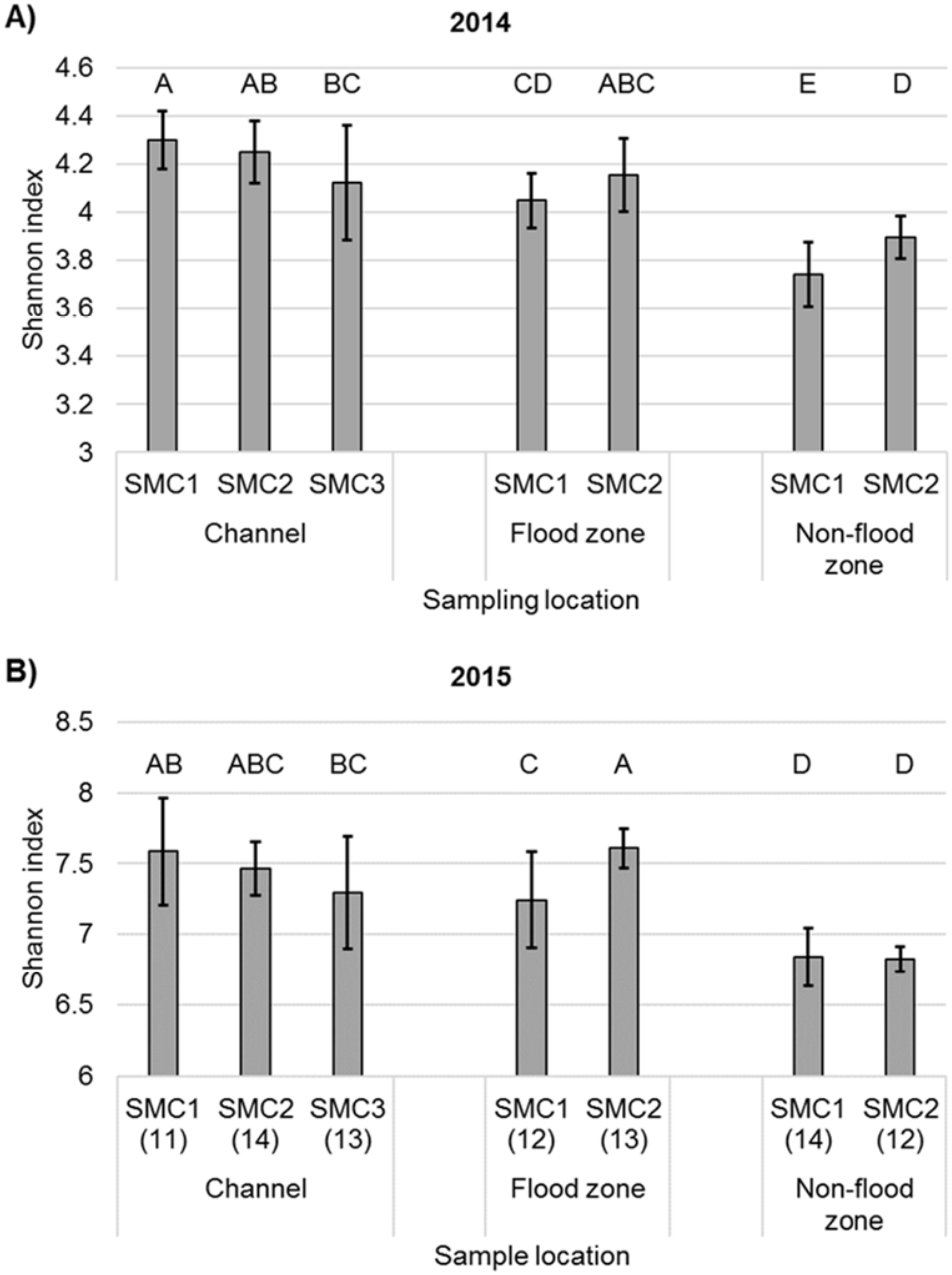

Figure S3 – Shannon indices among samples collected in A) 2014 and B) 2015. Error bars reflect standard deviation. In 2014, *n* = 9 for all groups. The numbers of samples are shown in parentheses for 2015.

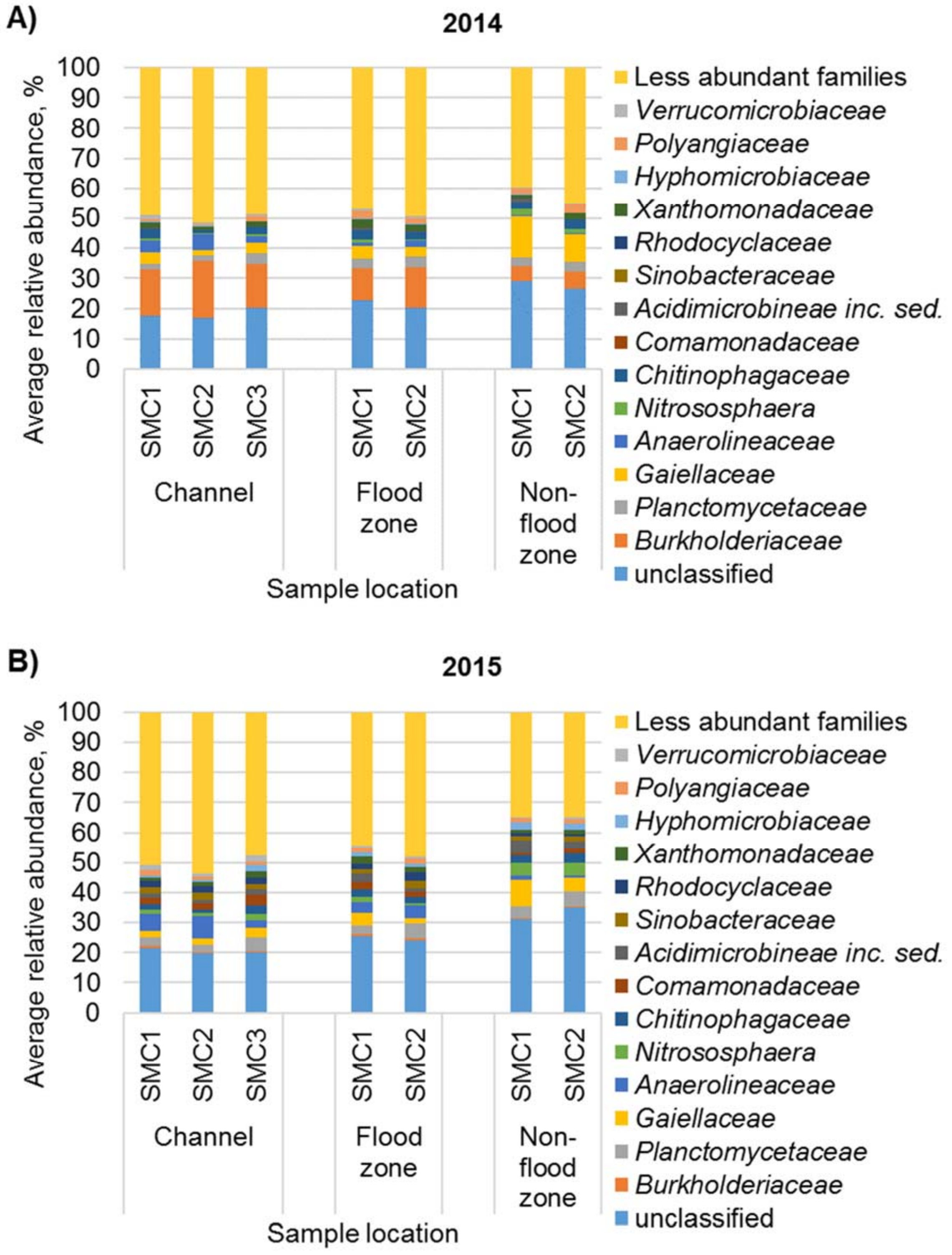

Figure S4 – Distribution of abundant families among samples collected in A) 2014 and B) 2015.

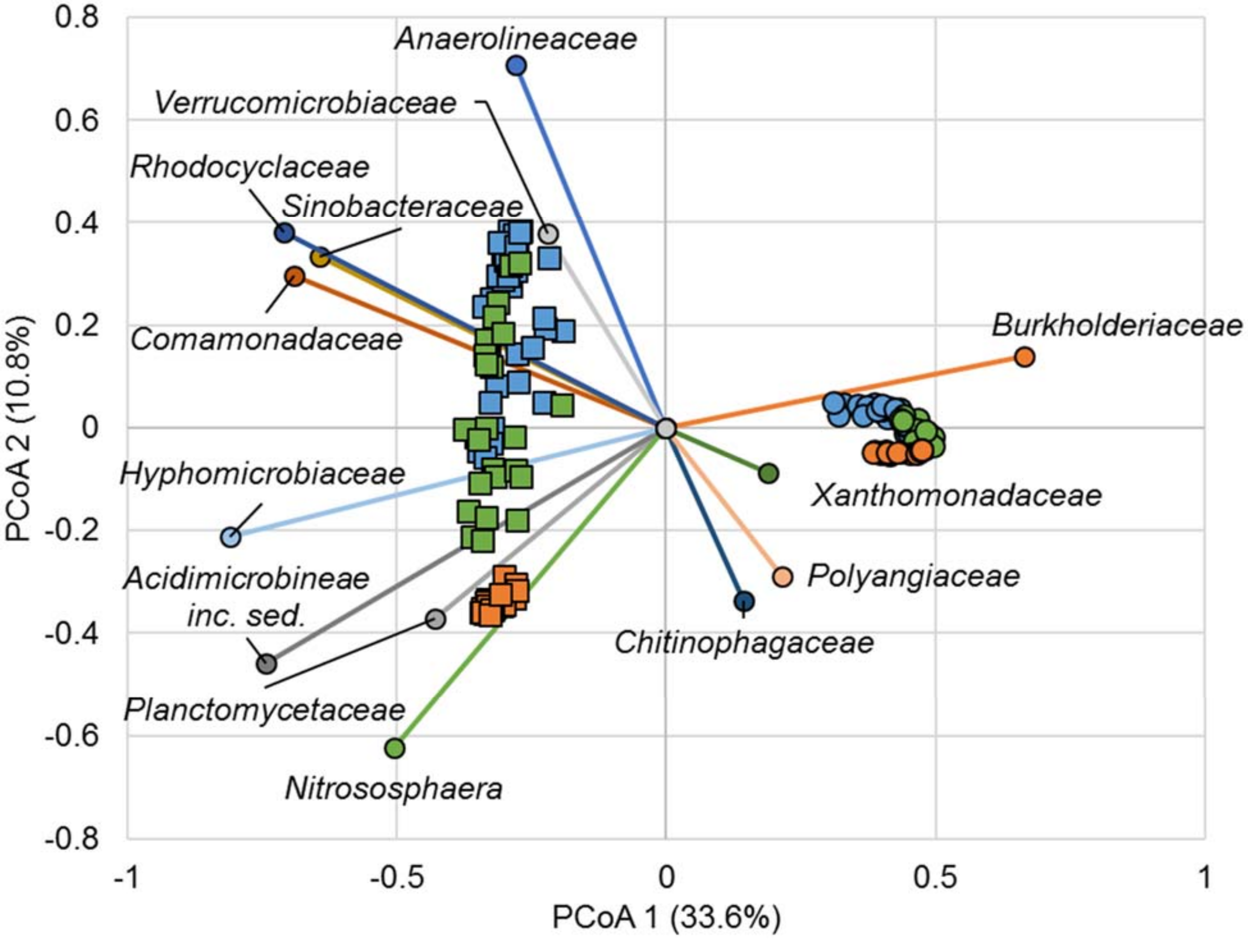

Figure S5. Principal coordinate analysis of Bray-Curtis dissimilarity matrices among samples collected during both years (r^2 = 0.83). Legend: 2014 (○), 2015 (□), channel (blue), flood zone (green), non-flood zone (orange). Families shown reflect predominant families (among the 15 most abundant) the abundance of which significantly affected ordination position (Spearman correlations, $P < 0.05$). Families that were not correlated with *x*- or *y*-axis positions are not shown.

Table S1 – Denitrification rates measured among samples collected in 2014. Mean and standard deviation are shown.

Date	Location	Site	DN_U (N_2O-N m^{-2} h^{-1})	DN_A (N_2O-N m^{-2} h^{-1})
12-Jun	Channel	SMC1	22.40 ± 19.25 A	23.96 ± 19.54 BCD
		SMC2	10.65 ± 7.70 AB	24.62 ± 12.80 BCD
		SMC3	0.51 ± 0.69 B	0.44 ± 0.42 F
	Flood zone	SMC1	7.26 ± 8.30 AB	41.38 ± 13.02 AB
		SMC2	15.62 ± 2.00 AB	49.27 ± 2.83 A
	Non-flood zone	SMC1	7.68 ± 2.16 AB	24.52 ± 2.73 BCD
		SMC2	7.97 ± 1.92 AB	18.37 ± 0.67 CDEF
20-Aug	Channel	SMC1	0.36 ± 0.27 B	6.18 ± 7.04 DEF
		SMC2	1.28 ± 0.27 B	37.82 ± 4.93 ABC
		SMC3	11.03 ± 1.50 AB	13.56 ± 1.13 DEF
	Flood zone	SMC1	9.33 ± 1.74 AB	26.40 ± 2.37 BCD
		SMC2	5.04 ± 1.78 B	42.15 ± 4.96 AB
	Non-flood zone	SMC1	5.69 ± 1.15 B	15.09 ± 0.94 DEF
		SMC2	6.55 ± 0.85 B	12.78 ± 0.38 DEF
20-Oct	Channel	SMC1	0.34 ± 0.17 B	15.58 ± 3.22 DEF
		SMC2	0.93 ± 0.13 B	23.63 ± 2.33 BCD
		SMC3	2.44 ± 3.35 B	2.46 ± 3.36 EF
	Flood zone	SMC1	7.17 ± 1.24 AB	22.34 ± 0.84 BCDE
		SMC2	4.02 ± 0.52 B	14.67 ± 1.78 DEF
	Non-flood zone	SMC1	9.09 ± 2.60 AB	26.01 ± 4.82 BCD
		SMC2	7.37 ± 2.15 AB	16.41 ± 0.93 DEF

$^{A,B,C,...}$ Values sharing the same letter did not differ significantly by Tukey's *post-hoc* test (P > 0.05).

Table S2 – Denitrification rates measured among samples collected in 2015. Mean and standard deviation are shown.

Date	Location	Site	DN_U (N_2O-N m^{-2} h^{-1})	DN_A (N_2O-N m^{-2} h^{-1})
12-May	Channel	SMC1	0.62 ± 0.07 F	18.39 ± 4.97 EFG
		SMC2	0.64 ± 0.13 F	16.32 ± 4.72 EFG
		SMC3	8.78 ± 2.38 EF	11.01 ± 4.90 EFG
	Flood zone	SMC1	0.86 ± 0.43 F	3.11 ± 1.42 G
		SMC2	3.00 ± 0.83 F	26.99 ± 5.49 DEFG
	Non-flood zone	SMC1	6.69 ± 2.81 EF	21.42 ± 2.81 EFG
		SMC2	10.22 ± 1.21 EF	16.74 ± 0.23 EFG
15-Jun	Channel	SMC1	31.55 ± 3.51 BCDE	42.26 ± 8.31 BCDEF
		SMC2	14.47 ± 2.81 DEF	19.84 ± 2.08 EFG
		SMC3	2.06 ± 1.61 F	1.29 ± 1.07 G
	Flood zone	SMC1	3.28 ± 3.24 F	8.04 ± 8.77 EFG
		SMC2	15.43 ± 2.04 CDEF	18.11 ± 1.05 EFG
	Non-flood zone	SMC1	10.90 ± 3.88 DEF	22.20 ± 3.44 DEFG
		SMC2	17.02 ± 6.61 CDEF	23.70 ± 9.46 DEFG
27-Jul	Channel	SMC1	4.52 ± 2.28 EF	22.02 ± 28.05 EFG
		SMC2	61.49 ± 21.42 A	103.11 ± 34.01 A
		SMC3	0.41 ± 0.36 F	1.26 ± 1.31 G
	Flood zone	SMC1	0.24 ± 0.08 F	3.29 ± 4.03 G
		SMC2	31.55 ± 25.78 BCDE	45.39 ± 13.89 BCDE
	Non-flood zone	SMC1	5.80 ± 3.03 EF	21.51 ± 3.14 EFG
		SMC2	6.44 ± 1.34 EF	18.28 ± 1.95 EFG
18-Aug	Channel	SMC1	19.89 ± 5.16 CDEF	26.38 ± 11.92 DEFG
		SMC2	22.64 ± 3.03 CDEF	27.85 ± 6.67 CDEFG
		SMC3	2.01 ± 1.24 F	1.73 ± 0.77 G
	Flood zone	SMC1	2.18 ± 2.28 F	6.27 ± 8.80 FG
		SMC2	42.41 ± 5.95 ABC	64.79 ± 16.26 BC
	Non-flood zone	SMC1	4.16 ± 0.92 EF	20.93 ± 4.87 EFG
		SMC2	8.74 ± 1.70 EF	18.69 ± 5.87 EFG
9-Nov	Channel	SMC1	14.57 ± 3.25 CDEF	13.33 ± 0.36 EFG
		SMC2	51.55 ± 31.69 AB	71.83 ± 37.23 AB
		SMC3	7.22 ± 7.77 EF	11.20 ± 3.35 EFG
	Flood zone	SMC1	4.61 ± 3.91 EF	4.50 ± 2.42 G
		SMC2	38.28 ± 5.42 ABCD	59.52 ± 4.20 BCD
	Non-flood zone	SMC1	11.54 ± 3.23 DEF	35.86 ± 3.93 BCDEFG
		SMC2	14.00 ± 7.47 DEF	33.07 ± 6.57 CDEFG

[A,B,C,...] Values sharing the same letter did not differ significantly by Tukey's *post-hoc* test ($P > 0.05$).

Table S3 – Physicochemical parameters measured among sampling sites. Mean and standard deviation are shown.

Year	Site	Location	Dry weight:wet weight	Volumetric water content (ml)	Bulk density (g cm^{-3})	Sediment organic matter (%)	Soil nitrate (mg NO_3-N /kg^{-1})
2014	SMC1	Channel	0.78 ± 0.02 AB	0.40 ± 0.04 CDEF	1.41 ± 0.09 AB	2.34 ± 0.70 D	0.45 ± 0.13 C
		Floodzone	0.67 ± 0.08 CD	0.42 ± 0.13 CDE	0.82 ± 0.05 EF	10.78 ± 1.19 BC	3.44 ± 1.76 BC
		Nonfloodzone	0.73 ± 0.04 BC	0.27 ± 0.05 F	0.71 ± 0.11 FG	17.56 ± 3.32 A	10.49 ± 5.9 B
	SMC2	Channel	0.65 ± 0.04 CDE	0.54 ± 0.03 BC	1.04 ± 0.14 DE	4.32 ± 0.83 D	0.36 ± 0.09 C
		Floodzone	0.60 ± 0.09 DE	0.44 ± 0.06 CDE	0.74 ± 0.13 FG	11.41 ± 3.13 BC	0.60 ± 0.19 C
		Nonfloodzone	0.74 ± 0.06 ABC	0.26 ± 0.10 F	0.73 ± 0.07 FG	13.48 ± 2.35 ABC	5.36 ± 2.59 BC
	SMC3	Channel	0.80 ± 0.04 AB	0.38 ± 0.06 DEF	1.5 0± 0.12 AB	1.64 ± 1.26 D	0.31 ± 0.06 C
2015	SMC1	Channel	0.73 ± 0.05 BC	0.49 ± 0.09 BCD	1.29 ± 0.12 BC	2.54 ± 1.02 D	0.53 ± 0.71 C
		Floodzone	0.70 ± 0.09 BC	0.50 ± 0.13 BCD	1.15 ± 0.22 CD	4.77 ± 2.77 D	6.74 ± 4.49 B
		Nonfloodzone	0.66 ± 0.03 CD	0.38 ± 0.05 EF	0.73 ± 0.11 FG	16.65 ± 2.34 A	24.16 ± 9.29 A
	SMC2	Channel	0.57 ± 0.12 E	0.59 ± 0.09 B	0.82 ± 0.28 F	5.52 ± 2.45 D	0.37 ± 0.26 C
		Floodzone	0.45 ± 0.07 F	0.77 ± 0.12 A	0.60 ± 0.10 G	9.64 ± 1.01 C	0.39 ± 0.17 C
		Nonfloodzone	0.66 ± 0.02 CD	0.43 ± 0.06 CDE	0.83 ± 0.10 EF	14.28 ± 4.25 AB	9.47 ± 7.79 B
	SMC3	Channel	0.82 ± 0.06 A	0.34 ± 0.11 EF	1.56 ± 0.16 A	2.22 ± 5.13 D	0.85 ± 1.19 C
		**Fisher's *F***	< 0.0001	< 0.0001	< 0.0001	< 0.0001	< 0.0001

[A,B,C,...] Values sharing the same letter did not differ significantly by Tukey's *post-hoc* test ($P > 0.05$).

Table S4 – Significant correlations (α = 0.05), determined by Spearman's rank correlation, between denitrification rates under site conditions (DN_U) and under non-limiting nutrient conditions (DN_A) and gene abundances for all sampling dates. Correlations that were not significant are not shown. NS: correlations were not significant.

		DN_U			DN_A		
		Channel	Flood zone	Non-flood zone	Channel	Flood zone	Non-flood zone
16S rRNA	ρ	0.490	0.640	-	0.781	0.608	-
	P-value	0.044	<0.001	NS	<0.001	0.010	NS
cnorB	ρ	0.693	0.889	-	0.671	0.866	-
	P-value	<0.001	<0.001	NS	<0.001	<0.001	NS
narG	ρ	-	0.775	-	0.656	0.794	-
	P-value	NS	<0.001	NS	<0.001	<0.001	NS
nirS	ρ	-	0.574	-	0.693	0.565	0.566
	P-value	NS	<0.001	NS	<0.001	0.028	0.026
nirK	ρ	0.707	0.762	-	0.678	0.748	-
	P-value	<0.001	<0.001	NS	<0.001	<0.001	NS
nosZ1	ρ	0.775	0.762	-	0.775	0.812	-
	P-value	<0.001	<0.001	NS	<0.001	<0.001	NS
nosZ3	ρ	0.600	0.800	-	0.800	0.781	-
	P-value	<0.001	<0.001	NS	<0.001	<0.001	NS
